# Supplementary material for: Hepatitis D virus infection triggers CXCL9-11 upregulation in hepatocytes and liver infiltration of CXCR3+ CD4 T cells
Source: JHEP Rep. 2024 Nov 14;7(3):101273. doi: 10.1016/j.jhepr.2024.101273 (PMC11840482; doi:10.1016/j.jhepr.2024.101273)
Supplement: Multimedia component 1 [file mmc1.pdf]

Hepatitis D Virus infection triggers CXCL9-11 upregulation in hepatocytes and liver infiltration of CXCR3+ CD4 T cells

Jan-Hendrik Bockmann, Lena Allweiss, Annika Volmari, David da Fonseca Araújo, Matin Kohsar, Anastasia Hyrina, Janine Kah, Zhijuan Song, Josolyn Chan, Katja Giersch, Tassilo Volz, Marc Lütgehetmann, Jeffrey J. Wallin, Dmitry Manuilov, Meghan M. Holdorf, Simon P. Fletcher, Ansgar W. Lohse, Antonio Bertoletti, Julian Schulze zur Wiesch, Maura Dandri

Table of contents

Fig. S1……………………………………………………………………………2

Fig. S2……………………………………………………………………………3

Fig. S3……………………………………………………………………………4

Fig. S4……………………………………………………………………………5

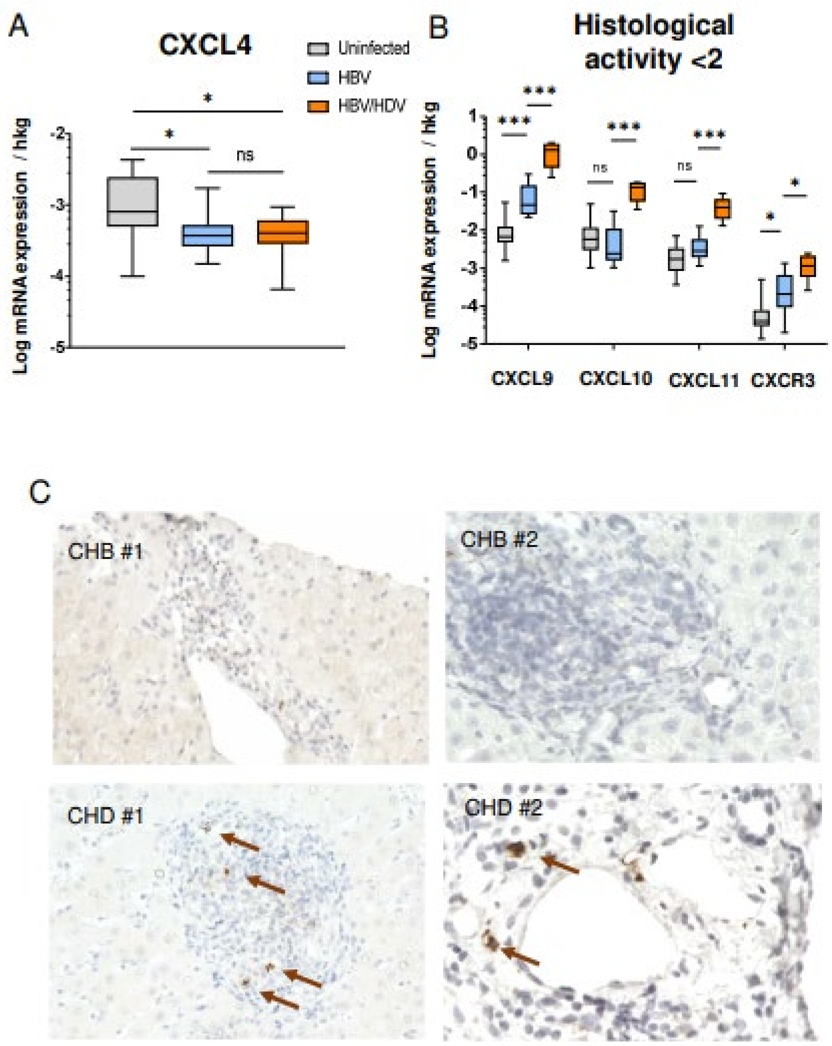

Fig. S1: (A) mRNA expression of CXCL4 analyzed by qPCR in patient liver biopsy samples. **(B)** mRNA expression of CXCR3 and corresponding chemokines analyzed by qPCR in liver biopsy samples from uninfected as well as CHB and CHD patients with low histological activity (<2). **(C)** IHC of CXCR3-positive cells in paraffin-embedded liver sections of CHD compared to CHB patients. Boxes represent interquartile range with median, vertical lines represent Min to Max errors, *p <0.05, ***p<0.001, ns, not significant (Mann-Whitney test).

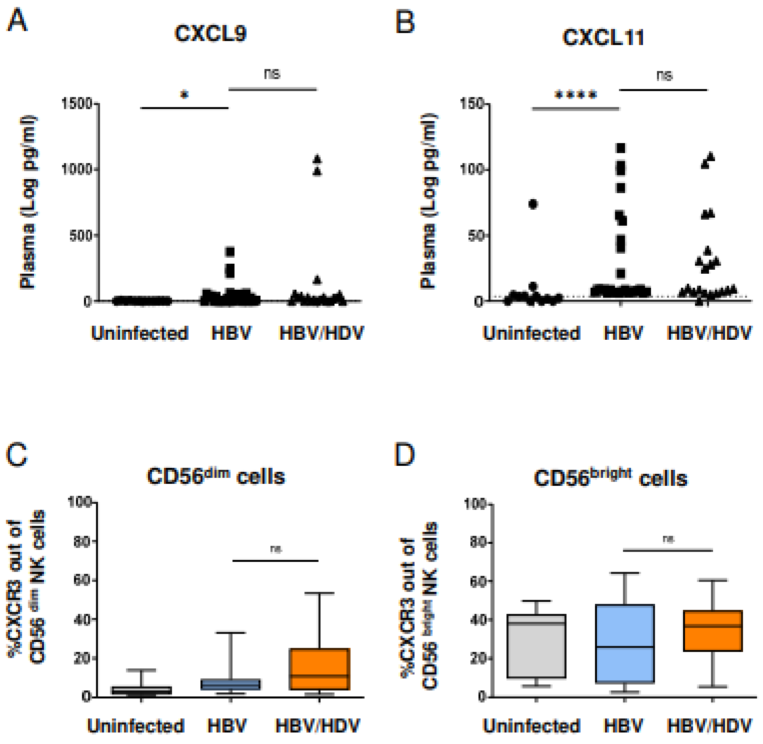

Fig. S2: Human **(A)** CXCL9 and **(B)** CXCL11 plasma protein levels and CXCR3-positive **(C)** CD56 dim NK cells, and **(D)** CD56 bright NK cells in patient blood samples analysed by Bead Conjugation Assay or FACS. Boxes represent interquartile range with median, vertical lines represent Min to Max errors, *p <0.05, ****p<0.0001, ns, not significant (Mann-Whitney test).

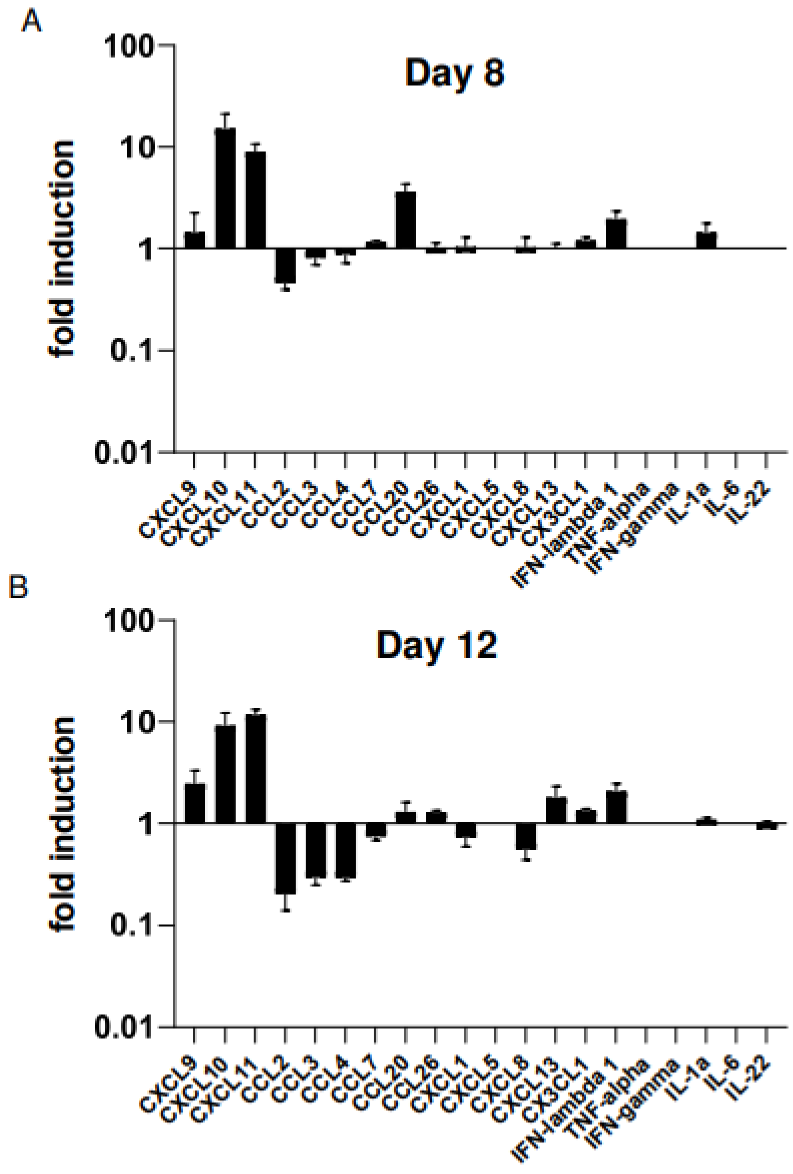

Fig. S3: Relative induction of secreted proteins in the supernatants of HDV mono-infected vs uninfected PHHs measured on the Luminex platform (**A)** 8 days and **(B**) 12 days after HDV infection. Boxes represent mean, vertical lines represent standard deviation.

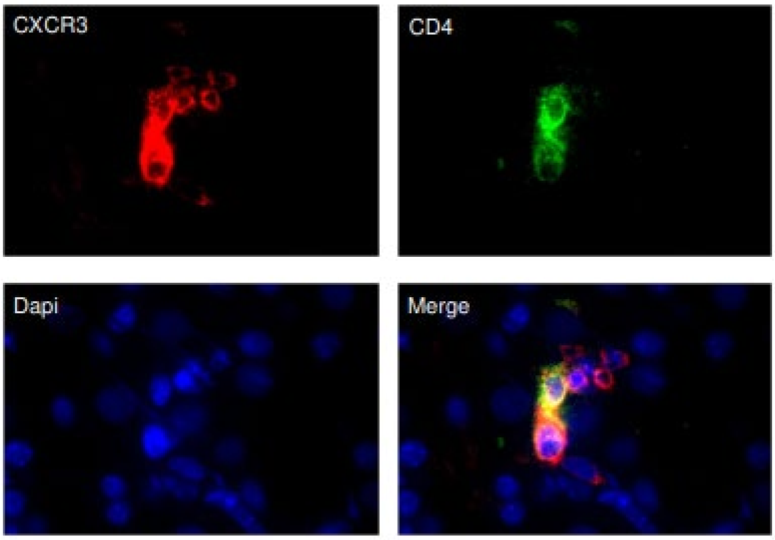

Fig. S4: CXCR3 (red)- and CD4 (green)- positive cells in frozen liver sections of HBV/HDV-infected chimeric mouse livers receiving HBsAg-specific T cells were analyzed by IF.
