# Supplementary material for: Hepatitis D virus infection triggers CXCL9-11 upregulation in hepatocytes and liver infiltration of CXCR3+ CD4 T cells
Source: JHEP Rep. 2024 Nov 14;7(3):101273. doi: 10.1016/j.jhepr.2024.101273 (PMC11840482; doi:10.1016/j.jhepr.2024.101273)
Supplement: Multimedia component 2 [file mmc2.docx]

**Journal of Hepatology**

**CTAT methods**

Tables for a “Complete, Transparent, Accurate and Timely account” (CTAT) are now mandatory for all revised submissions. The aim is to enhance the reproducibility of methods.

- Only include the parts relevant to your study
- Refer to the CTAT in the main text as ‘Supplementary CTAT Table’
- Do not add subheadings
- Add as many rows as needed to include all information
- Only include one item per row

**If the CTAT form is not relevant to your study, please outline the reasons why:**

|  |
| --- |

- 1. **Antibodies**

| **Name** | **Citation** | **Supplier** | **Cat no.** | **Clone no.** |
| --- | --- | --- | --- | --- |
| anti-HDAg antibody |  | Gilead | - | FD3A7 |
| Alexa Fluor-647-conjugated goat anti-mouse secondary antibody |  | Thermo Fisher | A-21235 | Polyclonal |
| Rabbit anti-human CD4 |  | Cell Signaling | 93518S | D2E6M |
| Mouse anti-human CD183 (CXCR3) |  | RD | MAB160-SP | 49801 |
| FITC anti-human CD183 (CXCR3) Antibody |  | Biolegend | 353704 | G025H7 |
| BUV737 Mouse Anti-Human CD56 |  | BD Biosciences | 564448 | NCAM16.2 |
| PerCP/Cyanine5.5 anti-human CD69 |  | Biolegend | 310926 | FN50 |
| PE anti-human CD154 |  | Biolegend | 310806 | 24-31 |
| BV 510 anti-human CD3 |  | Biolegend | 300448 | UCHT1 |
| PerCP/Cyanine5.5 anti-human CD4 |  | Biolegend | 344607 | SK3 |
| BUV737 Mouse Anti-Human CD4 |  | BD Biosciences | 564305 | SK3 |
| BV 421 anti-human CD197 (CCR7) |  | Biolegend | 353208 | G043H7 |
| BV 650 anti-human CD45RA |  | Biolegend | 304136 | HIT100 |

- 1. **Cell lines**

| **Name** | **Citation** | **Supplier** | **Cat no.** | **Passage no.** | **Authentication test method** |
| --- | --- | --- | --- | --- | --- |
| None |  |  |  |  |  |

- 1. **Organisms**

| **Name** | **Citation** | **Supplier** | **Strain** | **Sex** | **Age** | **Overall n number** |
| --- | --- | --- | --- | --- | --- | --- |
| USG mice |  | Maura Dandri | Urokinase-type plasminogen activator (uPA)/severe combined immunodeficiency(scid)/beige/  interleukin-2 receptor gamma chain negative (IL2Rγ-/-) mice |  |  | 36 |

- 1. **Sequence based reagents**

| **Name** | **Sequence** | **Supplier** |
| --- | --- | --- |
| CXCL9 | Hs00171065_m1 | Thermo Fisher |
| CXCL10 | Hs00171042_m1 | Thermo Fisher |
| CXCL11 | Hs00171138_m1 | Thermo Fisher |
| CXCR3 | Hs00171041_m1 | Thermo Fisher |
| CD4 | Hs01058407_m1 | Thermo Fisher |
| TBX21 | Hs00203436_m1 | Thermo Fisher |
| CXCL9 | assay number: 440161 | RNAscope, Bio-Techne |
| CXCL10 | assay number: 311851 | RNAscope, Bio-Techne |
| CXCR3 | assay number: 539251 | RNAscope, Bio-Techne |

- 1. **Biological samples**

| **Description** | **Source** | **Identifier** |
| --- | --- | --- |
| PHH | BioReclamation IVT | Cat# F00995-P |
| Liver biopsies from chronic hepatitis D patients registered in MYR202 trial | Gilead Sciences | NCT03546621 |
| PBMCs and liver biopsies from chronic hepatitis B/D patients and uninfected individuals | University Medical Center Hamburg-Eppendorf | PV5661 and PV4081 |

- 1. **Deposited data**

| **Name of repository** | **Identifier** | **Link** |
| --- | --- | --- |
| none |  |  |

- 1. **Software**

| **Software name** | **Manufacturer** | **Version** |
| --- | --- | --- |
| GraphPad Prism | GraphPad Software | 9.2.0 |
| BioRender | Biorender.com | - |
| FlowJo | BD | - |

- 1. **Other (e.g. drugs, proteins, vectors etc.)**

| HBsAg | Prospec | HBV-231 |
| --- | --- | --- |
| HDAg | Prospec | HDV-234 |

- 1. **Please provide the details of the corresponding methods author for the manuscript:**

| Jan-Hendrik Bockmann  University Medical Center Hamburg-Eppendorf  Martinistr. 52, D - 20246 Hamburg, Germany  Tel.: + 49 - 40 - 7410 55662, Fax: + 49 - 40 - 7410 57232  Email: j.bockmann@uke.de |
| --- |

**2.0 Please confirm for randomised controlled trials all versions of the clinical protocol are included in the submission. These will be published online as supplementary information.**

| Not applicable. |
| --- |
