# Supplementary material for: Hepatitis D virus infection triggers CXCL9-11 upregulation in hepatocytes and liver infiltration of CXCR3+ CD4 T cells
Source: JHEP Rep. 2024 Nov 14;7(3):101273. doi: 10.1016/j.jhepr.2024.101273 (PMC11840482; doi:10.1016/j.jhepr.2024.101273)
Supplement: Multimedia component 4 [file mmc4.pdf]

Hepatitis D virus infection triggers CXCL9-11 upregulation in hepatocytes and liver infiltration of CXCR3+ CD4 T cells

Jan-Hendrik Bockmann[1,2,*], Lena Allweiss[1,2], Annika Volmari[1,2], David da Fonseca Araújo[1], Matin Kohsar[1,3], Anastasia Hyrina[4], Janine Kah[1,5], Zhijuan Song[4], Josolyn Chan[4], Katja Giersch[1,6], Tassilo Volz[1,2], Marc Lütgehetmann[2,6], Jeffrey J. Wallin[4], Dmitry Manuilov[4], Meghan M. Holdorf[4], Simon P. Fletcher[4], Ansgar W. Lohse[1,2], Antonio Bertoletti[7], Julian Schulze zur Wiesch[1,2], Maura Dandri[1,2]

JHEP Reports **2025**. vol. 7 | 1–11

Check for updates

Background & Aims: The role of hepatocytes in producing chemokines and triggering liver inflammation and damage in chronic hepatitis D (CHD) is not fully understood. Herein, we investigated the contribution of primary human hepatocytes (PHHs) infected with HDV in triggering inflammation by producing the chemokines CXCL9–11.

Methods: We performed quantitative PCR, RNA *in situ* hybridisation, activation-induced marker (AIM) assays, and FACS analysis to investigate the CXCR3/CXCL9–11 receptor/ligand axis of T cells in peripheral blood and livers from patients with chronic hepatitis B (n = 27 and 18, respectively) and CHD (n = 20 and 18, respectively). Chemokine expression was investigated in cultured HDV-infected PHHs and in livers of HBV- or HBV/HDV-infected humanised mice in the presence or absence of adoptively transferred human immune cells (n = 35 in total).

Results: In patient and chimeric mouse livers, higher expression levels of CXCL9–11 were found in an HBV/HDV-coinfected *vs.* HBV-mono-infected setting. Similarly, high levels of CXCL9–11 were observed in HDV-infected PHHs *in vitro*. Analysis by RNA *in situ* hybridisation on patient livers revealed that HDV-infected hepatocytes were a significant contributor to the chemokine expression. The corresponding chemokine receptor CXCR3 was found upregulated specifically on peripheral bulk CD4 T cells of patients with CHD. CXCR3 upregulation was unspecific and was not detected on HDAg- or HBsAg-specific CD4 T cells by activation-induced marker assay. Lastly, adoptive transfer of human T cells in humanised mice led to recruitment of non-HBV/HDV-specific CD4+ T cells only in the setting of HBV/HDV coinfection, but not in HBV-mono-infected mice.

Conclusions: HDV infection upregulated the intrahepatic expression of the CXCL9–11/CXCR3 receptor/ligand axis. Higher amounts of HBV/HDV-unspecific CD4 T cells expressing CXCR3 may contribute to the aggravated liver inflammation frequently observed in patients with CHD.

© 2024 The Authors. Published by Elsevier B.V. on behalf of European Association for the Study of the Liver (EASL). This is an open access article under the CC BY license (http://creativecommons.org/licenses/by/4.0/).

Introduction

Approximately 12 million people are estimated to be chronically infected with both HBV and HDV worldwide.[1] Compared with chronic hepatitis B (CHB), HBV/HDV chronic infection (CHD) is associated with higher rates of liver cirrhosis and hepatocellular carcinoma (HCC).[2–4] Furthermore, CHD remains difficult to treat despite the availability of pegylated interferon-alpha (IFN-α) and the approval of the HBV/HDV entry inhibitor Hepcludex (bulevirtide/Myrcludex-B).

Apart from being a difficult-to-treat chronic hepatitis virus infection, little is known about the similarities and differences in the chemokine patterns and molecular mechanisms contributing to liver disease in HDV/HBV infection compared with HBV mono-infection. In CHB, CXCL10 has been described to correlate with advanced liver fibrosis and a favourable treatment response to IFN-α and nucleos(t)ide (NA) therapy.[5,6] We previously described higher inductions of human interferon-stimulated genes and CXCL10 in the livers of HBV/HDV-infected humanised mice compared with HBV-mono-infected animals.[7]

CXCL10, as well as CXCL9 and CXCL11, belong to the CXC chemokine family, all of which interact with the CXCR3 receptor expressed on the cell surface of several immune cell types, such as CD4 and CD8 T cells, natural killer (NK) cells, and B cells. Because these chemokines regulate the infiltration of CXCR3-expressing immune cells to sites of liver injury, they play a crucial role in various liver diseases, such as hepatitis C, metabolic dysfunction-associated steatohepatitis (MASH), and HCC.[8–11] Nevertheless, the ability of HDV to promote chemokine expression changes in the liver of patients with CHD and the potential impact that such changes may have on liver-resident and infiltrating immune cells, in particular on CXCR3-expressing immune cells, are poorly defined.

This study aimed to investigate chemokine patterns and their significance on CXCR3 expression and chemotaxis in the

* Corresponding author. Address: Department of Internal Medicine, Center for Internal Medicine, University Medical Center Hamburg-Eppendorf, Martinistr. 52, D-20246 Hamburg, Germany. Tel.: +49 40 7410 55662; fax: +49 40 7410 58531.
E-mail address: j.bockmann@uke.de (J.-H. Bockmann).
https://doi.org/10.1016/j.jhepr.2024.101273

livers of patients with CHD and CHB and in relevant *in vitro* and *in vivo* infection models, such as primary human hepatocytes (PHHs) and human liver chimeric mice. Human liver chimeric mice lack immune cells, such as T, B, and NK cells, but human immune cells can be adoptively transferred.[12,13] To dissect the cellular source and levels of distinct chemokines and their receptors, we performed both protein and transcriptional analyses, including single-cell visualisation, using *in vitro* and *in vivo* infection experiments, as well as comparative analyses of liver biopsies from uninfected and infected patients with CHB and CHD.

Patients and methods

Patients

In the current study, we collected patient plasma samples from 27 individuals with CHB, 20 with CHD, and 14 who were uninfected. Furthermore, we analysed liver biopsy samples obtained from 14 uninfected patients, 18 patients with CHB, and 18 patients with CHD (as part of the MYR202 clinical trial, molecular analyses of baseline biopsies).[14,15] The inclusion criteria were HBsAg positivity in patients infected with HBV and HBV/HDV and anti-HDV positivity in patients with CHD for at least 6 months. Patients who had undergone liver transplantation or showed a history of HCC or HIV/HCV coinfection were excluded from the study. Patient characteristics are shown in Tables 1 and 2.[14] Analyses were performed on peripheral blood mononuclear cells (PBMCs) and residual histopathological material from patients with CHB/CHD and healthy individuals (ethics numbers PV5661 and PV4081) and conformed to German law and the principles espoused in the Declaration of Helsinki. Informed consent was obtained from all participants.

In vitro experiments

For HDV mono-infection experiments, cryopreserved PHHs (BioIVT) were seeded onto collagen I-coated 96-well plates and maintained in William's E medium (containing 2% FBS and 1.5% DMSO). PHH supernatants were collected on days 1, 4, 6, 8, and 12 after HDV infection, and protein concentrations were measured using an immune monitoring 65-plex human ProcartaPlex panel (Thermo Fisher catalogue number EPX650-10065-901) and a custom 9-plex human ProcartaPlex panel that included IFN-α, IFN-γ, IL-29, IFN-β, IFN-ω, IL-1α, IL-6, CXCL10, and TNF-α (Thermo Fisher) on the Luminex FLEXMAP 3D instrument, following the manufacturer's protocol. Between days 1 and 13 after HDV infection, PHHs were fixed with 4% paraformaldehyde and stained using an anti-HDAg antibody (Clone FD3A7) and Alexa Fluor-647-conjugated goat anti-mouse secondary antibody (Thermo Fisher catalogue number A-21235). Cells were imaged using the CellInsight CX7 Pro HCS Platform (Thermo Fisher).

For HDV superinfection experiments, human PHHs were isolated from at least 12 weeks stably HBV (genotype D)-infected chimeric USG mouse livers by perfusion and collagenase digestion, followed by repeated low-speed centrifugations.[16] PHHs were seeded on collagen-coated 24-well plates and infected with HDV (genotype 1, multiplicity of infection = 1) 4 h after seeding. Cells were cultured in William's E medium (containing 5% FBS and 1.8% DMSO) and lysed 3, 7, and 14 days after infection for RNA isolation using a Qiagen RNeasy Kit following the manufacturer's protocol. Human intracellular RNA was analysed as described below. Analysis was performed using one PHH donor with three replicates.

For the activation-induced marker (AIM) assay, cryopreserved PBMCs were thawed, washed with RPMI, and plated on 96-well plates (1 × 10^6 cells per well). Cells were treated with HDAg, HBsAg, or the cytomegalovirus (CMV) peptide pool overnight (5 μg/ml) or were left unstimulated. Cells were then stained for 1 h at 4 °C with FACS antibodies and fixed with 1% paraformaldehyde. FACS analysis was performed on the same day. CD40L and CD69 were used as activation markers.

Mouse experiments

Human liver chimeric mice were generated as previously described.[16] Human hepatocytes from a single human donor were intrasplenically injected into 3-week-old USG (uPA/SCID/beige/IL2r$\gamma^{-/-}$) mice anesthetised with isoflurane. Human chimerism levels were determined by measuring human serum albumin in mouse serum using the Human Albumin ELISA kit (Immunology Consultants Lab, Portland, OR, USA). Successfully repopulated mice were infected with HBV (genotype D) or HBV and HDV (genotype 1) by a single intraperitoneal injection.[7,13] Stably infected mice were sacrificed after 8–12 weeks of infection. As previously reported, some mice were adoptively transferred using human CD4 and CD8 T cells that were engineered (via RNA electroporation) to express either HBsAg-specific or unspecific T-cell receptors (TCRs).[12,13] All animal experiments and procedures were performed in accordance with the European Union directive 86/609/EEC and approved by the ethical committee of the city and state of Hamburg in accordance with the principles of the Declaration of Helsinki.

Table 1. Patient characteristics of liver samples.

	HBV (n = 18)	HBV/HDV (n = 18)
Cirrhosis, n (%)	1 (5.56)	8 (44.44)
Age (years), median (IQR)	47 (38.5–53)	43 (38.25–46)
Male sex, n (%)	10 (55.56)	13 (72.22)
Treatment, n (%)	0 (0)	Tenofovir: 3 (16.67)
Grade of histological activity	0 (0–1) (Desmet)	1.5 (1–2) (METAVIR)
Stage of liver fibrosis	2 (1–2) (Desmet)	3 (1–2) (Ishak)
HBsAg (IU/ml), median (IQR)	4.6 × 10^3 (5.0 × 10^2-1.6 × 10^4)	1.2 × 10^4 (7.8 × 10^3-2.1 × 10^4)
HBV DNA (IU/ml), median (IQR)	6.0 × 10^4 (5.5 × 10^2-7.75 × 10^6)	0 (0-3.5 × 10^1)
HDV RNA (IU/ml), median (IQR)	—	3.4 × 10^5 (1.0 × 10^5-1.0 × 10^6)
ALT (U/L), median (IQR)	74 (42.7–122.2)	98.5 (58.5–144.7)

ALT, alanine aminotransferase.

Table 2. Patient characteristics of PBMC and plasma samples.

	HBV (n = 27)	HBV/HDV (n = 20)
Cirrhosis, n (%)	4 (14.8)	8 (40)
Age (years), median (IQR)	43 (32–46)	47 (38–57)
Male sex, n (%)	14 (51.8)	13 (65.0)
Treatment	History of NA: 7 (25.9)	History of NA: 7 (35.0)
HBeAg positive, n (%)	3 (11.11)	3 (15)
HBsAg (IU/ml), median (IQR)	7.6×10^3 (1.1×10^3-2.0×10^4)	7.06×10^3 (7.3×10^2-1.1×10^4)
HBV DNA (IU/ml), median (IQR)	1.5×10^3 (4.4×10^2-9.5×10^3)	4.2×10^2 (1.5×10^2-2.9×10^3)
HDV RNA (IU/ml), median (IQR)	—	5.2×10^4 (1.4×10^4-8.9×10^4)
Albumin (g/L), median (IQR)	40 (39–42)	38 (36–41)
INR, median (IQR)	1.00 (0.97–1.05)	1.1 (1.05–1.13)
Platelet count (10^9/L), median (IQR)	209 (162–241)	171 (129–206)
Bilirubin (mg/dl), median (IQR)	0.7 (0.4–0.8)	0.6 (0.4–0.8)
ALT (U/L), median (IQR)	34 (22–51)	69 (28–107)
AST (U/L), median (IQR)	25 (19–31)	46 (27–73)
GGT (U/L), median (IQR)	37 (24–96)	40.5 (25–66)

ALT, alanine aminotransferase; AST, aspartate aminotransferase; INR, international normalised ratio; NA, nucleos(t)ide analogue; PBMC, peripheral blood mononuclear cell; GGT, gamma-glutamyltransferase.

Quantitative PCR

Total RNA was isolated using the RNeasy Mini Kit (Qiagen) and was transcribed into cDNA using oligo-dT primers and the Transcriptor Kit (Roche Applied Science). Quantitative PCR (qPCR) was performed using human-specific Taqman Gene Expression Assays (Thermo Fisher). The analysed genes were human genes CXCL9 (Hs00171065_m1), CXCL10 (Hs00171042_m1), CXCL11 (Hs00171138_m1), CXCR3 (Hs00171041_m1), CD4 (Hs01058407_m1), and TBX21 (Hs00203436_m1).

Plasma chemokine measurements

Plasma from hepatitis patients was collected during clinical visits and stored at -80 °C. Quantification of chemokines was performed using a bead-based multiplex assay LEGENDPlex (BioLegend) in 96-well plates. Samples were measured using LSRFortessa (BD), and the acquired data were analysed using LEGENDPlex Software V8.

RNA *in situ* hybridisation (RNAScope)

RNA *in situ* hybridisation (ISH) was performed on formalin-fixed, paraffin-embedded liver sections using the RNAscope® 2.5 HD Duplex Assay (Bio-Techne, Minneapolis, MN, USA) according to the manufacturer's instructions and using human-specific target probes for human CXCL9 (assay number 440161), human CXCL10 (assay number 311851), and human CXCR3 (assay number 539251). Stained sections were analysed by fluorescence microscopy (Biorevo BZ-9000, Keyence, Osaka, Japan).

Software and statistical analysis

The graphical abstract was created with BioRender.com. Figures and statistics were performed using the GraphPad Prism 9 software (GraphPad Software Inc., San Diego, CA, USA). For comparisons of different patient or mouse cohorts, the nonparametric Mann–Whitney *U* test was applied. A value of $p < 0.05$ was considered statistically significant.

Results

CXCL9–11 are highly expressed by hepatocytes and correlate with CXCR3-expressing cell infiltrates in the livers of patients with CHD compared with patients with CHB

To investigate chemokine expression patterns in the setting of CHD and CHB, we determined the gene expression levels of the CXCR3 ligands in liver samples of 18 patients with CHB, 18 patients with CHD, and 14 uninfected controls. The clinical characteristics are shown in Table 1. CXCL9, 10, and 11 were significantly elevated in both infected groups compared with healthy controls (Fig. 1A). However, expression levels of CXCL9 (p <0.0001, 16.09-fold), CXCL10 (p <0.0001, 16.63-fold), and CXCL11 (p <0.0001, 9.59-fold) were significantly higher in the livers of patients with CHD compared with patients with patients with CHB. Compared with uninfected individuals, the strongest elevation was determined for CXCL9 in patients with CHD (p <0.0001, 207.33-fold) and CHB (p <0.0001, 12.89-fold). CXCL4, which can also bind to CXCR3, was not induced in the livers of patients with CHB or CHD compared with healthy individuals (Fig. S1A). Of note, although higher levels of intrahepatic inflammation were determined in the livers of patients with CHD compared with patients with CHB, significantly higher expression levels of CXCL9, 10, and 11, as well as CXCR3, could also be determined in patients with CHD displaying low histological activity compared with the livers of patients with CHB (Fig. S1B). Within patients with CHD, CXCL9–11 mRNA expression did not correlate significantly with histological activity (Metavir) or liver fibrosis (Ishak) using Spearman correlation.

In line with the qPCR data, RNA *in situ* visualisation (ISH) revealed substantial expression of CXCL9- and CXCL10-positive cells in HBV/HDV-coinfected compared with HBV-mono-infected or uninfected livers (Fig. 1B). Single-cell analysis of human *CXCL9* mRNA and HDV RNA performed via RNA ISH in liver biopsies from patients with CHD revealed co-expression of HDV and CXCL9 RNA in HBV/HDV-coinfected human liver biopsies (Fig. 1C), thus identifying hepatocytes as producers of these chemokines in human livers.

Of note, higher *CXCR3* mRNA expression was detected by qPCR in the livers of patients with CHD compared with those

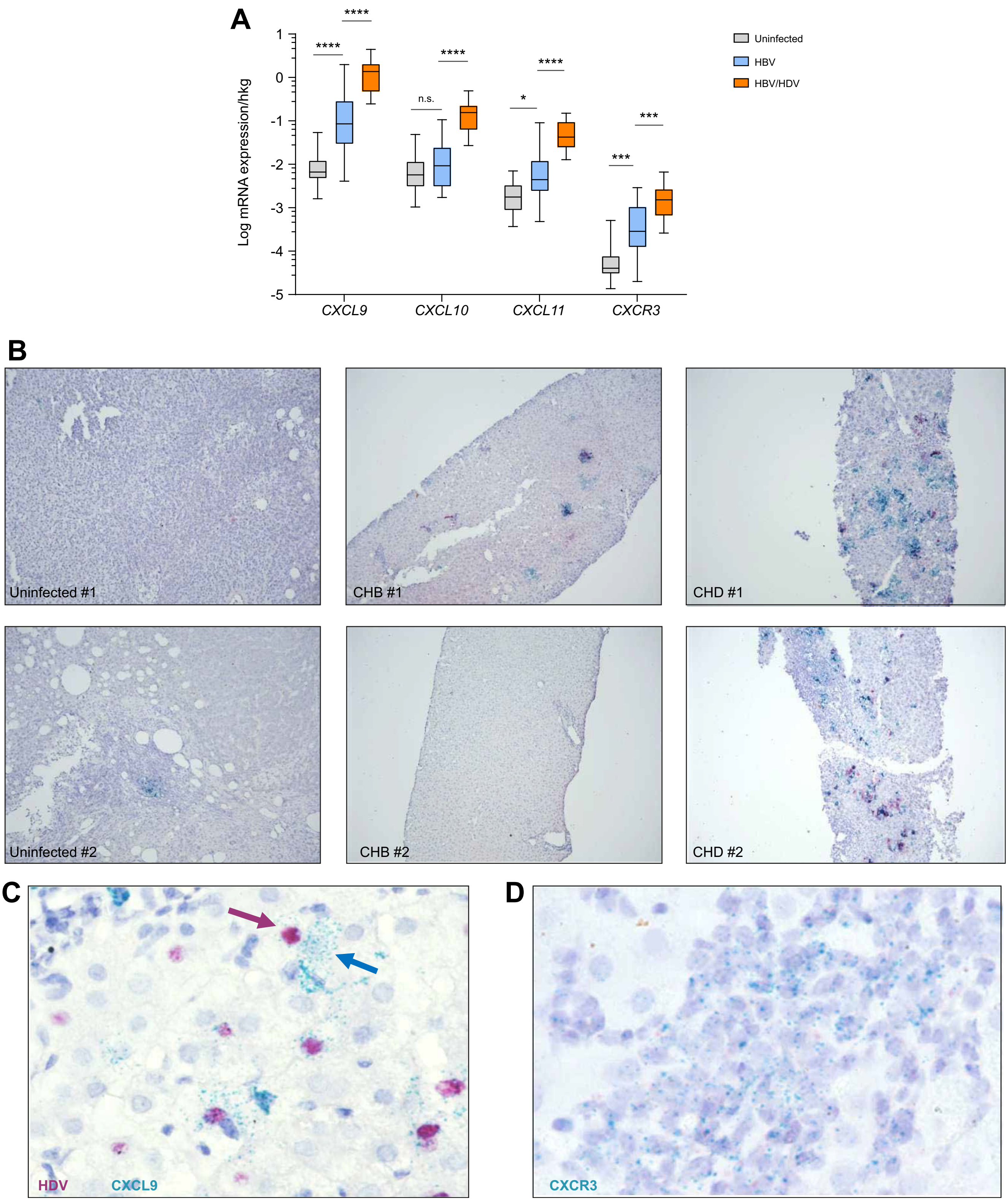

Fig. 1. HDV infection induces higher levels of CXCR3 and its chemokine ligands CXCL9–11 than HBV infection in patients. (A) mRNA expression of CXCR3 and corresponding chemokines analysed by qPCR in liver biopsy samples from uninfected patients (n = 14), patients with CHB (n = 18), and patients with CHD (n = 18). (B) A stronger CXCL9 expression was observed by RNA ISH in the livers of patients with CHD compared with CXCL10 expression. CXCL9 (turquoise) and CXCL10 (red) were determined in the livers of patients who were uninfected, with CHB, and with CHD. (C) RNA ISH of CXCL9 (turquois, arrows) in relation to HDV RNA (red) in liver tissues of patients with CHD. (D) RNA ISH of CXCR3 (turquoise) in CHD liver biopsy samples. Boxes represent IQR range with median, and vertical lines represent min to max errors. *p <0.05; ***p <0.001; ****p <0.0001; ns, not significant (Mann–Whitney U test). CHB, chronic hepatitis B; CHD, chronic hepatitis D; ISH, *in situ* hybridisation; qPCR, quantitative PCR.

with CHB (p <0.001, 5.30-fold), suggesting higher frequencies of CXCR3-expressing cells in livers of patients with CHD (Fig. 1A). Indeed, RNA ISH and immunohistochemistry analyses demonstrated higher CXCR3 expression within areas of immune cell infiltrates in HBV/HDV-coinfected patient livers than in HBV-mono-infected livers (Fig. 1D and Fig. S1C).

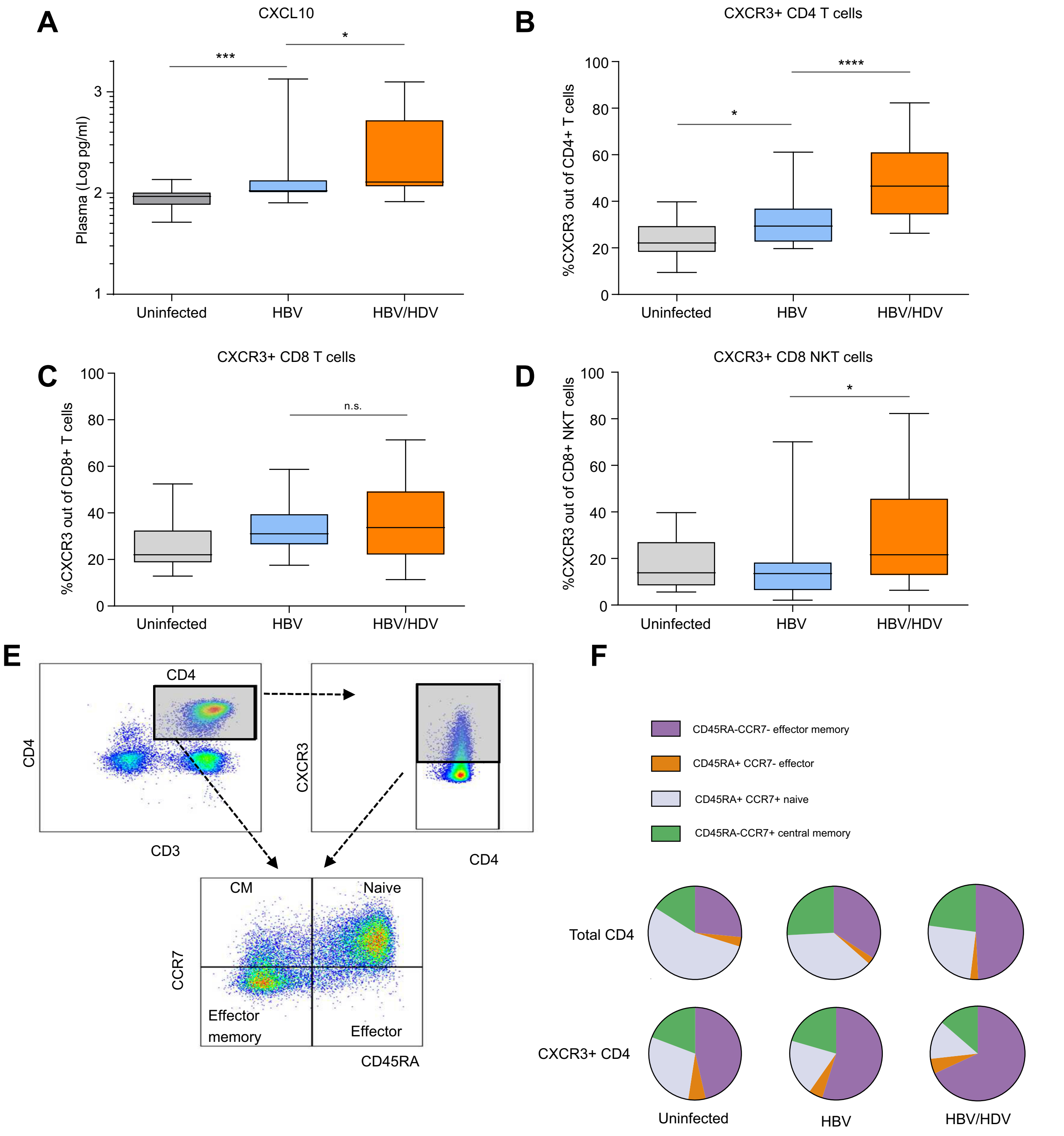

Fig. 2. CXCR3 is upregulated on peripheral CD4 T cells in patients with CHD. (A) Human CXCL10 protein levels, (B) CXCR3+ CD4 T cells, (C) CD8 T cells, and (D) CD8 NK T cells in blood samples of 14 patients who were uninfected, 27 with CHB, and 20 with CHD were determined by Bead Conjugation Assay or FACS analysis. (E) Gating strategy of CD4 memory T cell subtypes. (F) Frequency of memory T cells among total and CXCR3+ CD4 T cells. Boxes represent IQR range with median, and vertical lines represent min to max errors. *p <0.05; ***p <0.001; ****p <0.0001; ns, not significant (Mann–Whitney U test). CHB, chronic hepatitis B; CHD, chronic hepatitis D; NK, natural killer.

CXCR3 is upregulated on CD4+ T cells, defining the predominant TH1-like memory phenotype in the periphery of patients with CHD

Plasma levels of CXCL10 and CXCR3 expression on PBMCs were analysed in 27 patients with CHB and 20 patients with CHD (see characteristics in Table 2). CXCL10 protein levels were slightly, but significantly, higher in patients with CHD than in patients with CHB (p = 0.0366, 1.22-fold) and uninfected healthy controls (p <0.0001, 1.39-fold) (Fig. 2A). In sharp contrast to the intrahepatic situation, CXCL9 and CXCL11 plasma protein levels did not differ between patients with CHB and CHD (Fig. S2A and B). However, the median frequency of CXCR3+CD4+ T cells was higher in blood samples of patients with CHD than in patients with CHB (p <0.0001; 46.57% *vs.* 29.37%) (Fig. 2B). Moreover, CXCR3+ CD8+ NK T cells were slightly induced in patients with CHD, whereas the frequency of CXCR3+ NK and CD8 T cells did not differ significantly between patients with CHB and CHD (Fig. 2C and D and Fig. S2C and D). Subtype analysis revealed that in CHD, CXCR3+CD4+ T cells were mainly effector memory T cells (CD4+CXCR3+CD45RA-CCR7-; 68.4%) (Fig. 2E and F).

To compare CXCR3 expression between different virus-specific T cells, PBMCs from individuals with CHB or CHD were stimulated with either HBsAg or HDAg, whereas CMV pp65 protein was used as the reference viral antigen. The co-expression of AIMs CD40L and CD69 was then analysed by FACS to quantify antigen-specific CD4 T cells (Fig. 3A). Stimulation with the indicated antigens detected significantly higher (relative to unstimulated) frequencies of HDAg-specific CD4 T cells in patients with CHD compared with HBsAg- and pp65-specific T cells in patients with CHD or CHB (Fig. 3B). Interestingly, phenotype analysis of total and antigen-specific CD4 T cells showed that the frequencies of HDAg-specific and HBsAg-

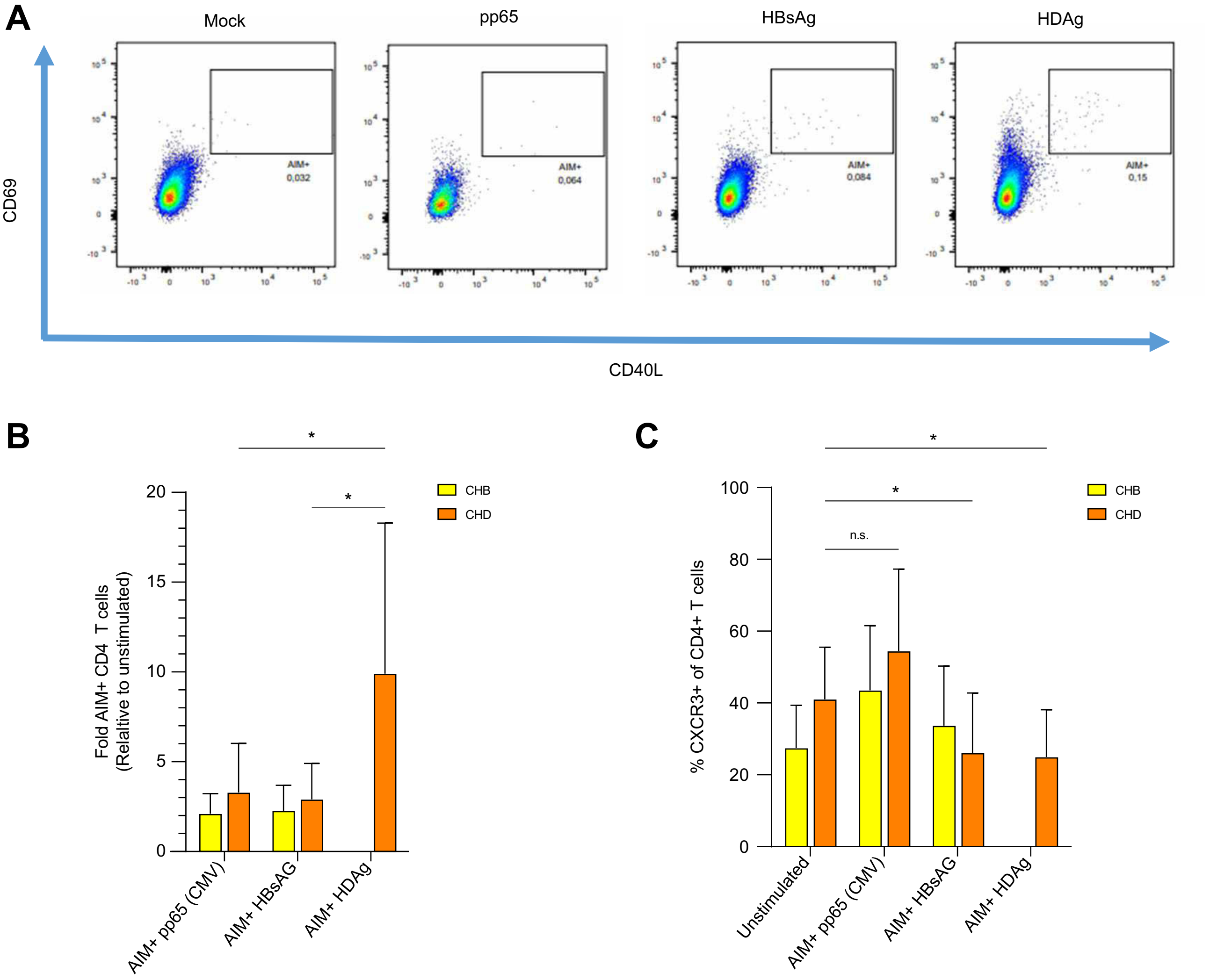

Fig. 3. CXCR3 is upregulated on total but not on HBV/HDV-specific CD4 T cells. (A, B) PBMCs of 13 patients with CHB and 12 patients with CHD were stimulated overnight with antigens pp65 (CMV), HDAg (HDV), and HBsAg (HBV). Antigen-specific CD4 T cells were identified by co-expression of AIM-markers CD40L and CD69. (C) Frequencies of CXCR3+ cells were analysed on total (unstimulated) as well as pp65-, HBsAg-, and HDAg-stimulated AIM+ CD4 T cells by FACS. Bars represent mean, and vertical lines represent standard deviation. *p <0.05, ns, not significant (Mann–Whitney U test). AIM, activation-induced marker; CHB, chronic hepatitis B; CHD, chronic hepatitis D; pp65, phosphoprotein 65.

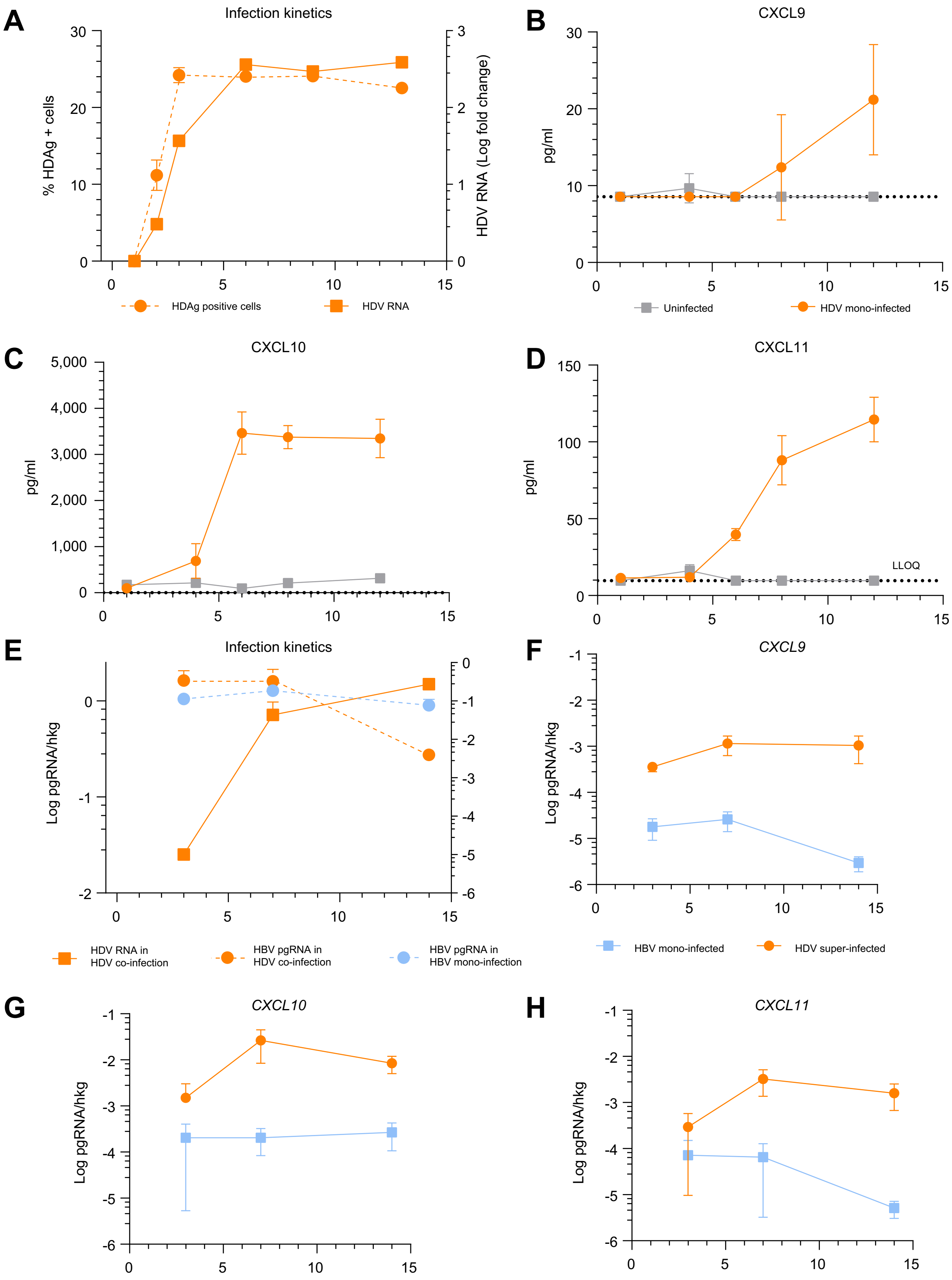

Fig. 4. HDV infection stimulates CXCL9–11 chemokine secretion in PHHs. (A) Kinetics of intracellular HDAg and HDV RNA following HDV mono-infection in PHHs. (B–D) Supernatants were collected on days 1, 4, 6, 8, and 12 post infection, and protein concentrations of (B) CXCL9, (C) CXCL10, and (D) CXCL11 were measured on the Luminex platform. (E) *In vitro* HDV superinfection of PHHs isolated from stably HBV-infected USG mouse livers. Intracellular HDV RNA and HBV pgRNA infection kinetics were determined in the presence or absence of HDV by qPCR. (F–H) Chemokine mRNA expression of *CXCL9–11* in infected cell cultures. Viral RNA and

specific CXCR3+ CD4 T cells were lower than total and pp65-specific CXCR3+ CD4 T cells in patients with CHD, whereas the frequencies of CXCR3+ CD4 T cells were similar between total, pp65-, and HBsAg-specific T cells in patients with CHB. This indicates that HBsAg- and HDAg-specific CD4 T cells in patients with CHD do not show significant CXCR3 upregulation contrary to total and CMV-specific T cells (Fig. 3C).

HDV infection induces high levels of CXCL9–11 in human hepatocytes *in vitro*

A broad range of cytokines and chemokines was also analysed in the supernatants of PHHs infected *in vitro* with HDV for up to 12 days. After 6 days of HDV mono-infection, steady-state levels of intracellular HDV RNA with 24% HDAg-positive cells were achieved (Fig. 4A). Although most chemokines/cytokines did not change or remained undetectable during stable infection on days 8 and 12 (Fig. S3), CXCL9, CXCL10, CXCL11 (Fig. 4B–D), and IFN-λ1 (Fig. S3) were increasingly induced at both time points post infection. In addition to IFN-λ1 expression that has been described to be elevated in CHD infection,[7] CXCR3 ligands CXCL9–11 appeared as those chemokines dominantly induced in stably HDV-mono-infected PHHs.

To investigate whether HDV alters the expression of CXCR3 ligands CXCL9, CXCL10, and CXCL11 upon superinfection of stably HBV-infected hepatocytes, we isolated PHHs from stably HBV-infected USG mouse livers and infected them with HDV *in vitro*. Superinfection resulted in increasing levels (2.73 $\times 10^4$-fold) of intracellular HDV RNA from days 3 to 14 after infection (Fig. 4E). Concomitantly, HBV pgRNA ($p = 0.033$, 5.95-fold) declined (Fig. 4E). CXCL9, CXCL10, and CXCL11 induction was higher in HDV-super-infected cells than in HBV-mono-infected controls, reaching the highest levels after 7 days of HDV superinfection (Fig. 4F–H).

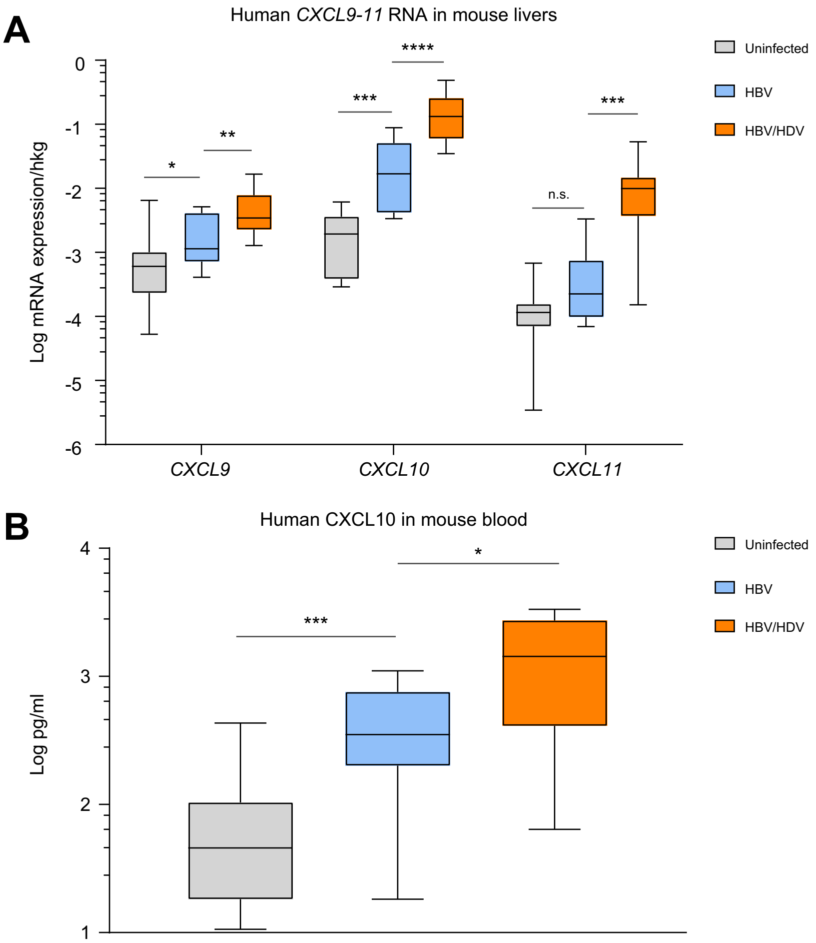

Fig. 5. Virus-mediated increase of human CXCL9–11 in human hepatocytes and sera of humanised mice. (A) Intrahepatic gene expression of chemokines *CXCL9–11* determined in human hepatocytes of uninfected, HBV, or HBV/HDV-coinfected USG mice. (B) Human CXCL10 protein levels in sera of mice determined using a Bead Conjugation Assay. Boxes represent IQR range with median, and vertical lines represent min to max errors. *p <0.05; **p <0.01; ***p <0.001; ****p <0.0001; ns, not significant (Mann–Whitney U test).

chemokine gene expression were analysed by qPCR on days 3, 7, and 14 after HDV infection. Points represent mean, and vertical lines represent standard deviation. PHH, primary human hepatocyte; qPCR, quantitative PCR.

HDV infection induces higher levels of the CXCR3 ligands CXCL9–11 than HBV in human hepatocytes in immune-deficient chimeric mice

To assess the ability of HDV to induce chemokines *in vivo* and to analyse the major cell types involved in CXCR3 ligand expression, we determined chemokine expression in human liver chimeric mice that were infected either with HBV or with HBV and HDV using species-specific qPCR probes recognising human cDNA. In line with the *in vitro* data, human CXCL9 (p = 0.009, 3.03-fold), CXCL10 (p <0.0001, 7.82-fold), and CXCL11 (p = 0.0002, 43.89-fold) were significantly elevated in human hepatocytes of HBV/HDV-coinfected mice compared with HBV-mono-infected mouse livers (Fig. 5A). Although plasma levels of circulating human CXCL9 and CXCL11 were partly not detectable, human CXCL10 protein levels were significantly elevated in HBV/HDV-coinfected mouse serum samples compared with HBV-mono-infected (p = 0.0278, 4.07-fold) and uninfected mice (p = 0.0005, 31.34-fold) (Fig. 5B), thus demonstrating the ability of human hepatocytes to contribute to chemokine production in response to HDV infection.

HDV/HDV coinfection is associated with higher levels of CXCR3 expression in humanised mouse livers upon adoptive T cell transfer

Recruitment of CXCR3-expressing lymphocytes and NK cells to sites of liver inflammation by the CXCL9–11 chemotaxis has been proposed for several liver diseases.[8,10] Because we observed particularly high CXCL9–11 expression in patients with CHD and in HBV/HDV-coinfected livers from humanised mice, we wondered if such an intrahepatic milieu could contribute to the recruitment of CXCR3-expressing infiltrates.

We previously demonstrated the antiviral efficacy of engineered human T cells expressing HBsAg-specific TCRs to recognise HBV-infected hepatocytes in both HBV- and HBV/HDV-infected chimeric mice.[12,13] To explore whether HDV infection may also favour the hepatic recruitment of antigen-specific and eventually also of non-antigen-specific immune cells expressing CXCR3, we comparatively analysed a cohort of HBV-mono-infected or HDV-super-infected mice that had been adoptively transferred with blood-derived human T cells expressing either an HBV-specific TCR or an infection-unrelated TCR.[12,13] Intrahepatic human mRNA expression of CXCR3 and Th1 cell transcription factor T-bet (TBX21) was enhanced upon transfer of HBV-specific T cells in both HBV-mono-infected and HBV/HDV dually infected animals. Human T cells that did not express HBV-specific TCRs were not recruited to HBV-mono-infected livers. Intriguingly, not only HBV-specific but also non-antigen-specific T cells were found in the livers of HBV/HDV-infected mice. Accordingly, human CXCR3, T-bet, and CD4 mRNA levels were enhanced in HBV/HDV-infected but not in HBV-mono-infected livers that received non-HBV-specific T cells (Fig. 6A–D). Furthermore, immunofluorescence co-staining of human CXCR3 and CD4 demonstrated CXCR3-positive CD4 T cells in HBV/HDV-infected livers receiving HBV-unspecific or HBV-specific T cells (Fig. S4). Altogether, these results show that HDV infection triggers the induction of the CXCR3/CXCL9–11 axis, and hence of a higher inflammatory milieu, able to promote intrahepatic recruitment of T cells independent of antigen-specific T cell recognition.

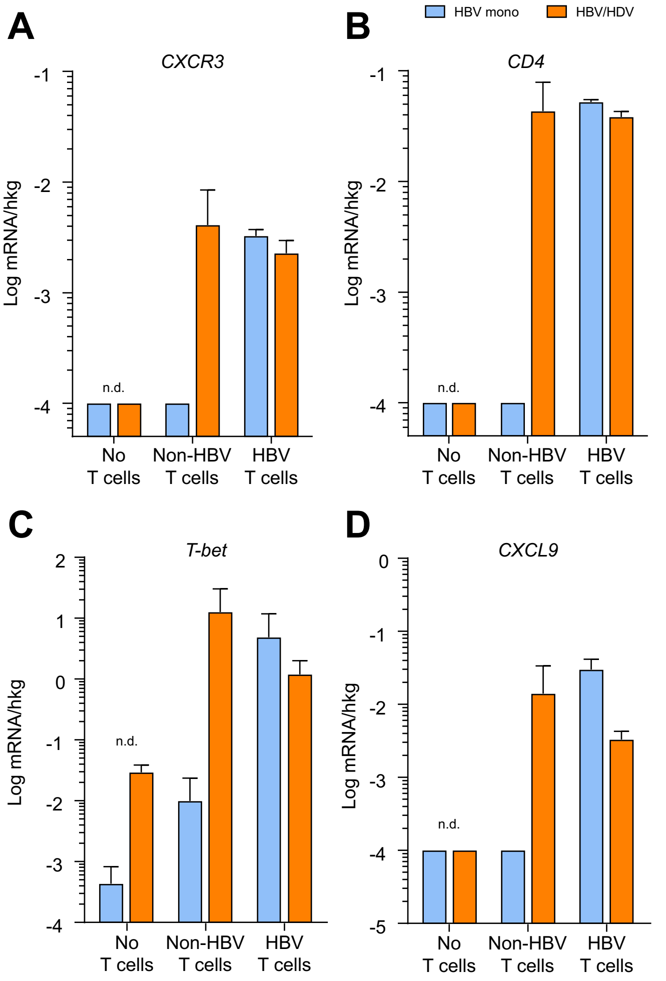

Fig. 6. Intrahepatic expression of human Th1 CD4 T-cell markers and ligands in HBV- and HBV/HDV-infected USG mouse livers in response to adoptive transfer of human immune cells. mRNA expression of (A) *CXCR3*, (B) *CD4*, (C) *T-bet*, and (D) *CXCL9* in HBV-mono-infected and HBV/HDV-coinfected chimeric livers without and after adoptive transfer of T cells expressing either HBV-nonspecific or HBV-specific T cell receptors. Bars represent mean, and vertical lines represent standard deviation. n.d., not detectable.

Discussion

Hepatocytes can secrete a broad repertoire of signalling molecules and chemokines. Alterations in the hepatocyte transcriptional setting, as those described during MASH progression, were shown to alter the intrahepatic hepatocyte-immune cell crosstalk.[17] Although the ability of hepatocytes to sense and signal the presence of viral infection is considered central to initiating and coordinating adequate immune responses to achieve infection control, the strict host and tissue tropism of human hepatitis viruses have hindered in-depth understanding of virus-mediated alterations of PHHs and whether virus-mediated changes may impact the immune responses, as well as liver disease.

CXCR3 ligands have been characterised as predictors of HBsAg reduction by IFN-α or NA treatment in HBV mono-infection[5,6] and are expressed at higher levels in HBV/HDV-coinfected hepatocytes in humanised mice and *in vitro*.[7,12,18] However, little is known about the role of the hepatocytes in chemokine production in the setting of HBV and HDV infection in

patients with CHD and whether hepatocyte-mediated alterations of the intrahepatic milieu can impact the immune responses. Most previous studies have focused on the analysis of peripheral chemokines. Townsend *et al.*[19] found higher ratios of Th1/Th2 cytokines in sera of patients with CHD compared with patients with CHB and healthy controls, an observation that pointed to more pronounced cellular immune responses in CHD. Other analyses of cytokine/chemokine profiles within cohorts with hepatitis D identified some cytokines and chemokines (*e.g.* CCL2 and CCL27) to be decreased in patients with high hepatitis D viraemia compared with patients with low viraemia.[20] Moreover, Wranke *et al.*[21] showed a correlation between CXCL10 and anti-HDV IgM as a marker for disease activity, although CXCL10 did not correlate with hepatitis D viraemia.

Cytokine profile differences determined between HBV mono-infection and HBV/HDV coinfection in the blood of chronically infected individuals may be difficult to interpret for various reasons. Complex mechanisms of HBV/HDV–host interplay act in the liver, the main organ where differences in pathology can be detected. Certainly, both patient groups share one virus (HBV), but the coexistence of HDV and variable amounts of HBV and HDV infection often leads to virological interferences and fluctuations in hepatitis B and D viraemia.[22] Because HDV infection generally contributes to faster disease progression, this study aimed to assess whether HDV substantially contributes to chemokine production in hepatocytes by using both experimental systems and patient liver biopsies. Moreover, we explored whether the increase in CXCR3 ligands is associated with or may even promote the accumulation of CXCR3-expressing effector cells in the liver.

The pro-inflammatory chemokines CXCL9, CXCL10, and CXCL11 were the main chemokines within a broad panel of cytokines/chemokines analysed that appeared vigorously induced in both HDV-mono-infected and super-infected hepatocytes *in vitro* and *in vivo* in human hepatocytes of HBV/HDV-coinfected patients and chimeric mice compared with HBV-mono-infected livers. CXCL10 levels were also elevated in sera of HBV/HDV-coinfected mice and patients compared with HBV-mono-infected individuals, although CXCL9 and CXCL11 levels remained barely detectable in most serum samples, indicating that HDV-driven induction of these chemokines differs between the liver and the periphery and is more accentuated intrahepatically. Interestingly, RNA ISH analysis of patient liver biopsies indicated that CXCL9 induction was stronger than CXCL10 induction in HDV-infected livers. The role of CXCL9 remains controversial. Although some mouse models have proposed antifibrotic effects in murine livers,[23] recent patient studies have highlighted CXCL9 as a marker for progression towards end-stage liver disease.[24,25] Thus, a vigorous CXCL9 induction in CHD may represent an important feature of HDV-induced immune pathogenesis.

Because HDV-coinfected livers express markedly higher levels of all three CXCR3 ligands CXCL9–11 in hepatocytes, chemotaxis of CXCR3-expressing cells may be expected. Indeed, we observed higher levels of CXCR3-expressing infiltrates in livers of HDV-infected patients. In line with patient data, enhanced mRNA expression of CXCR3 and the presence of CXCR3+ CD4 T cells were also detected in HBV/HDV-infected mice receiving not-virus-specific T cells, whereas only an HBV-specific recruitment of engineered T cells was determined in HBV-mono-infected mice. Although a strong CXCR3 upregulation was exclusively detected on CD4 T cells in the periphery of patients with CHD, CXCR3 might be upregulated on other immune cells intrahepatically. A limitation of this study was, therefore, the lack of intrahepatic FACS analysis needed to assess CD4 T cell co-expression markers. Furthermore, because od the limited sample size, we could not detect a correlation between CXCR3 ligands and HDV RNA expression or liver inflammation. However, a substantial reduction of CXCR3 receptors and ligands was recently shown in the liver of patients with CHD receiving bulevirtide treatment. Interestingly, the decrease of CXCR3 ligands correlated with HDV RNA and alanine aminotransferase reductions observed under bulevirtide treatment. Altogether these analyses indicate that the CXCL9–11/CXCR3 receptor ligand axis plays an important role in HDV-mediated liver inflammation.[15]

The potential role of bystander antigen-unspecific T cells in liver disease progression is gaining increasing recognition. Liver-resident and auto-aggressive CD8 T cells have been highlighted as key players in MASH immunopathology,[26] and even in CHB and CHD, liver-resident bystander CD8 T cells have been shown to correlate with liver inflammation.[27–29] Thus far, the activation and liver infiltration of Th1-polarised CD4 T cells has been reported for autoimmune hepatitis.[30] Here, we describe for the first time the capacity of HDV to enhance chemotaxis of Th1-polarised virus-unspecific CD4 T cells to the liver. Because CHD is the most severe form of viral hepatitis, both the previously reported capacity of HDV to trigger an upregulation of MHC molecules at the surface of the hepatocytes[12,31] and the enhanced recruitment of Th1-polarised virus-unspecific CD4+ T cells to the liver may be central to aggravate the liver disease course and warrant further investigation.

Affiliations

[1]*Department of Internal Medicine, University Medical Center Hamburg-Eppendorf, Hamburg, Germany;* [2]*German Center for Infection Research (DZIF), Hamburg-Lübeck-Borstel-Riems site, Germany;* [3]*Center for Tropical Medicine, Bernhard Nocht Institute for Tropical Medicine, Hamburg, Germany;* [4]*Gilead Sciences, Foster City, CA, USA;* [5]*Center of Internal Medicine II, Brandenburg Medical School Theodor Fontane, Brandenburg an der Havel, Germany;* [6]*Department of Medical Microbiology, Virology and Hygiene, University Medical Center Hamburg-Eppendorf, Hamburg, Germany;* [7]*Emerging Infectious Disease Program, Duke-NUS Medical School, Singapore*

Abbreviations

AIM, activation-induced marker; CHB, chronic hepatitis B; CHD, chronic hepatitis D; CMV, cytomegalovirus; CXCL9/10/11, C-X-C motif chemokine ligand 9/10/11; CXCR3, C-X-C motif chemokine receptor 3; HCC, hepatocellular carcinoma; IFN-α, interferon-alpha; ISH, *in situ* hybridisation; MASH, metabolic dysfunction-associated steatohepatitis; NA, nucleos(t)ide analogue; NK, natural killer; PBMC, peripheral blood mononuclear cell; PHH, primary human hepatocyte; pp65, phosphoprotein 65; qPCR, quantitative PCR; TCR, T-cell receptor.

Financial support

AWL, MD, ML, and JSzW were supported by the German Research Foundation (DFG) within the SFB841. JSzW is supported by the DFG SFB1328. JHB, LA, MD,

ML, and JSzW are funded by the German Center for Infection Research (DZIF: TI 07.002; TTU-Hepatitis 05.820; 05.822; 05.714). MD has research collaboration with Gilead.

Conflicts of interest

JHB and LA have nothing to declare. MD is an advisor for Gilead and Aligos. AH, ZS, JC, JJW, DM, MMH, and SPF are employees of Gilead Sciences. Inc.

Please refer to the accompanying ICMJE disclosure forms for further details.

Authors' contributions

Concept and design: JHB, ML, SPF, AWL, AB, MD, JSZW. Experiments and procedures: JHB, LA, AV, DFA, MK, AH, JK, ZS, JC, KG, TV, JJW, DM, MMH. Writing—original draft preparation: JHB, LA, MD, JSZW.

Data availability statement

The datasets used and/or analysed during the current study are available from the corresponding author upon reasonable request.

Acknowledgements

We thank Corinna Eggers and Martina Fahl for the great technical help.

Supplementary data

Supplementary data to this article can be found online at https://doi.org/10.1016/j.jhepr.2024.101273.

References

Author names in bold designate shared co-first authorship

[1] Stockdale AJ, Kreuels B, Henrion MYR, et al. The global prevalence of hepatitis D virus infection: systematic review and meta-analysis. J Hepatol 2020;73:523–532.
[2] Buti M, Homs M, Rodriguez-Frias F, et al. Clinical outcome of acute and chronic hepatitis delta over time: a long-term follow-up study. J Viral Hepat 2011;18:434–442.
[3] Farci P, Niro GA. Clinical features of hepatitis D. Semin Liver Dis 2012;32:228–236.
[4] Sureau C, Negro F. The hepatitis delta virus: replication and pathogenesis. J Hepatol 2016;64:S102–S116.
[5] Sonneveld MJ, Arends P, Boonstra A, et al. Serum levels of interferon-gamma-inducible protein 10 and response to peginterferon therapy in HBeAg-positive chronic hepatitis B. J Hepatol 2013;58:898–903.
[6] **Jaroszewicz J**, **Ho H**, Markova A, et al. Hepatitis B surface antigen (HBsAg) decrease and serum interferon-inducible protein-10 levels as predictive markers for HBsAg loss during treatment with nucleoside/nucleotide analogues. Antivir Ther 2011;16:915–924.
[7] Giersch K, Allweiss L, Volz T, et al. Hepatitis delta co-infection in humanized mice leads to pronounced induction of innate immune responses in comparison to HBV mono-infection. J Hepatol 2015;63:346–353.
[8] Zhang X, Han J, Man K, et al. CXC chemokine receptor 3 promotes steatohepatitis in mice through mediating inflammatory cytokines, macrophages and autophagy. J Hepatol 2016;64:160–170.
[9] Wasmuth HE, Lammert F, Zaldivar MM, et al. Antifibrotic effects of CXCL9 and its receptor CXCR3 in livers of mice and humans. Gastroenterology 2009;137:309–319.e3.
[10] Wasmuth HE, Tacke F, Trautwein C. Chemokines in liver inflammation and fibrosis. Semin Liver Dis 2010;30:215–225.
[11] Elia G, Fallahi P. Hepatocellular carcinoma and CXCR3 chemokines: a narrative review. Clin Ter 2017;168:e37–e41.
[12] Tham CYL, Kah J, Tan AT, et al. Hepatitis delta virus acts as an immunogenic adjuvant in hepatitis B virus-Infected hepatocytes. Cell Rep Med 2020;1:100060.
[13] Kah J, Koh S, Volz T, et al. Lymphocytes transiently expressing virus-specific T cell receptors reduce hepatitis B virus infection. J Clin Invest 2017;127:3177–3188.
[14] Wedemeyer H, Schoneweis K, Bogomolov P, et al. Safety and efficacy of bulevirtide in combination with tenofovir disoproxil fumarate in patients with hepatitis B virus and hepatitis D virus coinfection (MYR202): a multicentre, randomised, parallel-group, open-label, phase 2 trial. Lancet Infect Dis 2023;23:117–129.
[15] Allweiss L, Volmari A, Suri V, et al. Blocking viral entry with bulevirtide reduces the number of HDV-infected hepatocytes in human liver biopsies. J Hepatol 2024;80:882–891.
[16] Allweiss L, Volz T, Giersch K, et al. Proliferation of primary human hepatocytes and prevention of hepatitis B virus reinfection efficiently deplete nuclear cccDNA in vivo. Gut 2018;67:542–552.
[17] **Loft A**, **Schmidt SF**, **Caratti G**, et al. A macrophage-hepatocyte glucocorticoid receptor axis coordinates fasting ketogenesis. Cell Metab 2022;34:473–486.e9.
[18] Chida T, Ishida Y, Morioka S, et al. Persistent hepatic IFN system activation in HBV-HDV infection determines viral replication dynamics and therapeutic response. JCI Insight 2023;8:e162404.
[19] Townsend EC, Zhang GY, Ali R, et al. The balance of type 1 and type 2 immune responses in the contexts of hepatitis B infection and hepatitis D infection. J Gastroenterol Hepatol 2019;34:764–775.
[20] Lutterkort GL, Wranke A, Hengst J, et al. Viral dominance patterns in chronic hepatitis delta determine early response to interferon alpha therapy. J Viral Hepat 2018;25:1384–1394.
[21] Wranke A, Heidrich B, Ernst S, et al. Anti-HDV IgM as a marker of disease activity in hepatitis delta. PLoS One 2014;9:e101002.
[22] Dandri M, Volmari A, Lutgehetmann M. The hepatitis delta virus and chronic hepatitis D. J Hepatol 2022;77:1448–1450.
[23] Sahin H, Borkham-Kamphorst E, Kuppe C, et al. Chemokine Cxcl9 attenuates liver fibrosis-associated angiogenesis in mice. Hepatology 2012;55:1610–1619.
[24] **Berres ML**, **Asmacher S**, Lehmann J, et al. CXCL9 is a prognostic marker in patients with liver cirrhosis receiving transjugular intrahepatic portosystemic shunt. J Hepatol 2015;62:332–339.
[25] Tacke F, Zimmermann HW, Berres ML, et al. Serum chemokine receptor CXCR3 ligands are associated with progression, organ dysfunction and complications of chronic liver diseases. Liver Int 2011;31:840–849.
[26] Dudek M, Pfister D, Donakonda S, et al. Auto-aggressive $CXCR6^+$ CD8 T cells cause liver immune pathology in NASH. Nature 2021;592:444–449.
[27] Kefalakes H, Horgan XJ, Jung MK, et al. Liver-resident bystander $CD8^+$ T cells contribute to liver disease pathogenesis in chronic hepatitis D virus infection. Gastroenterology 2021;161:1567–1583.e9.
[28] Maini MK, Boni C, Lee CK, et al. The role of virus-specific $CD8^+$ cells in liver damage and viral control during persistent hepatitis B virus infection. J Exp Med 2000;191:1269–1280.
[29] Nkongolo S, Mahamed D, Kuipery A, et al. Longitudinal liver sampling in patients with chronic hepatitis B starting antiviral therapy reveals hepatotoxic CD8+ T cells. J Clin Invest 2023;133:e158903.
[30] Bovensiepen CS, Schakat M, Sebode M, et al. TNF-producing Th1 cells are selectively expanded in liver infiltrates of patients with autoimmune hepatitis. J Immunol 2019;203:3148–3156.
[31] Dandri M, Bertoletti A, Lutgehetmann M. Innate immunity in hepatitis B and D virus infection: consequences for viral persistence, inflammation, and T cell recognition. Semin Immunopathol 2021;43:535–548.

Keywords: HBV; HDV; CXCL10; CXCL9; CXCR3; Chemokines; CD4 T cell.
Received 19 January 2024; received in revised form 25 October 2024; accepted 8 November 2024; Available online 14 November 2024

Journal of Hepatology, Volume *7*

Supplemental information

Hepatitis D virus infection triggers CXCL9-11 upregulation in hepatocytes and liver infiltration of CXCR3+ CD4 T cells

Jan-Hendrik Bockmann, Lena Allweiss, Annika Volmari, David da Fonseca Araújo, Matin Kohsar, Anastasia Hyrina, Janine Kah, Zhijuan Song, Josolyn Chan, Katja Giersch, Tassilo Volz, Marc Lütgehetmann, Jeffrey J. Wallin, Dmitry Manuilov, Meghan M. Holdorf, Simon P. Fletcher, Ansgar W. Lohse, Antonio Bertoletti, Julian Schulze zur Wiesch, and Maura Dandri

Hepatitis D Virus infection triggers CXCL9-11 upregulation in hepatocytes and liver infiltration of CXCR3+ CD4 T cells

Jan-Hendrik Bockmann, Lena Allweiss, Annika Volmari, David da Fonseca Araújo, Matin Kohsar, Anastasia Hyrina, Janine Kah, Zhijuan Song, Josolyn Chan, Katja Giersch, Tassilo Volz, Marc Lütgehetmann, Jeffrey J. Wallin, Dmitry Manuilov, Meghan M. Holdorf, Simon P. Fletcher, Ansgar W. Lohse, Antonio Bertoletti, Julian Schulze zur Wiesch, Maura Dandri

Table of contents

Fig. S1……………………………………………………………………………2

Fig. S2……………………………………………………………………………3

Fig. S3……………………………………………………………………………4

Fig. S4……………………………………………………………………………5

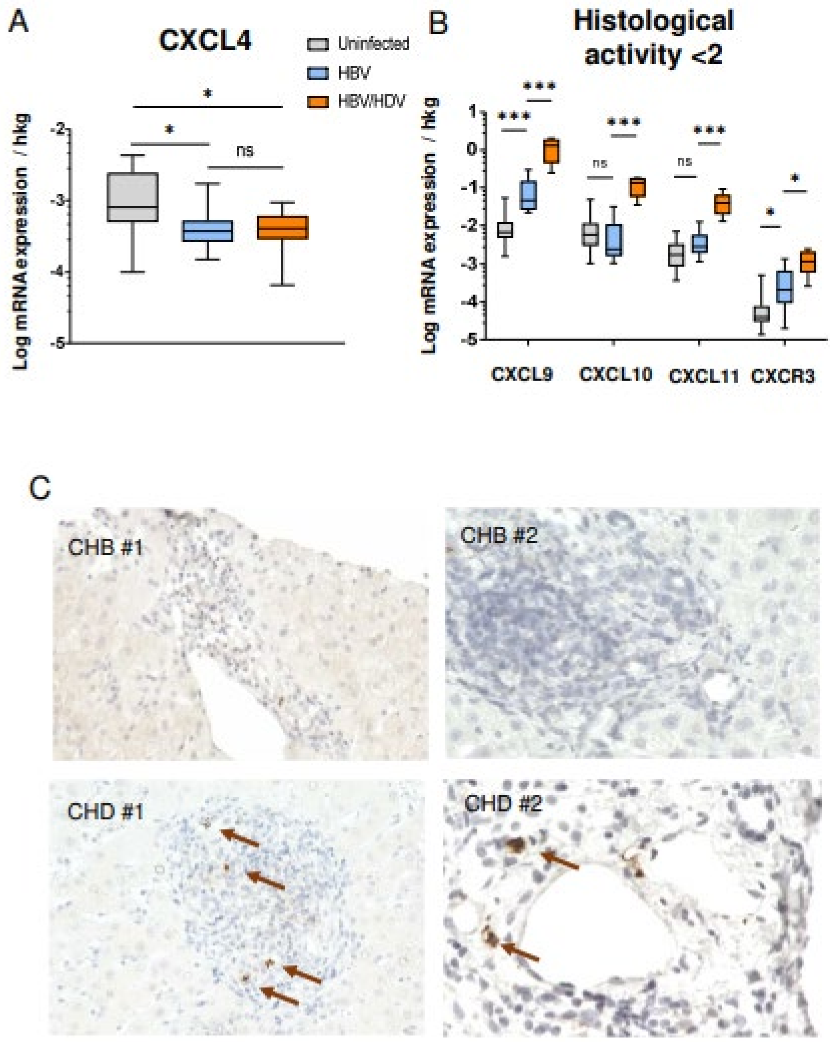

Fig. S1: (A) mRNA expression of CXCL4 analyzed by qPCR in patient liver biopsy samples. **(B)** mRNA expression of CXCR3 and corresponding chemokines analyzed by qPCR in liver biopsy samples from uninfected as well as CHB and CHD patients with low histological activity (<2). **(C)** IHC of CXCR3-positive cells in paraffin-embedded liver sections of CHD compared to CHB patients. Boxes represent interquartile range with median, vertical lines represent Min to Max errors, *p <0.05, ***p<0.001, ns, not significant (Mann-Whitney test).

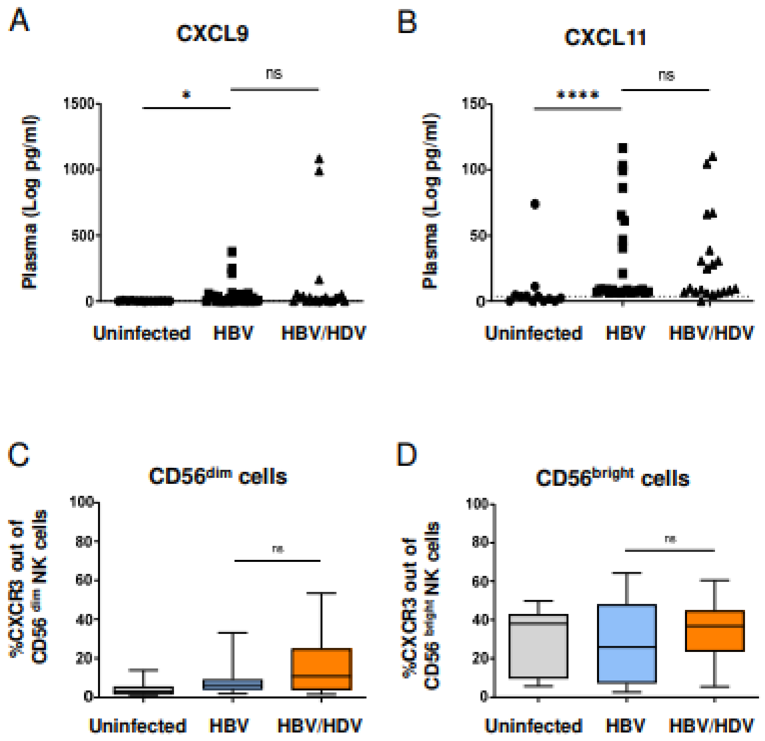

Fig. S2: Human **(A)** CXCL9 and **(B)** CXCL11 plasma protein levels and CXCR3-positive **(C)** CD56 dim NK cells, and **(D)** CD56 bright NK cells in patient blood samples analysed by Bead Conjugation Assay or FACS. Boxes represent interquartile range with median, vertical lines represent Min to Max errors, *p <0.05, ****p<0.0001, ns, not significant (Mann-Whitney test).

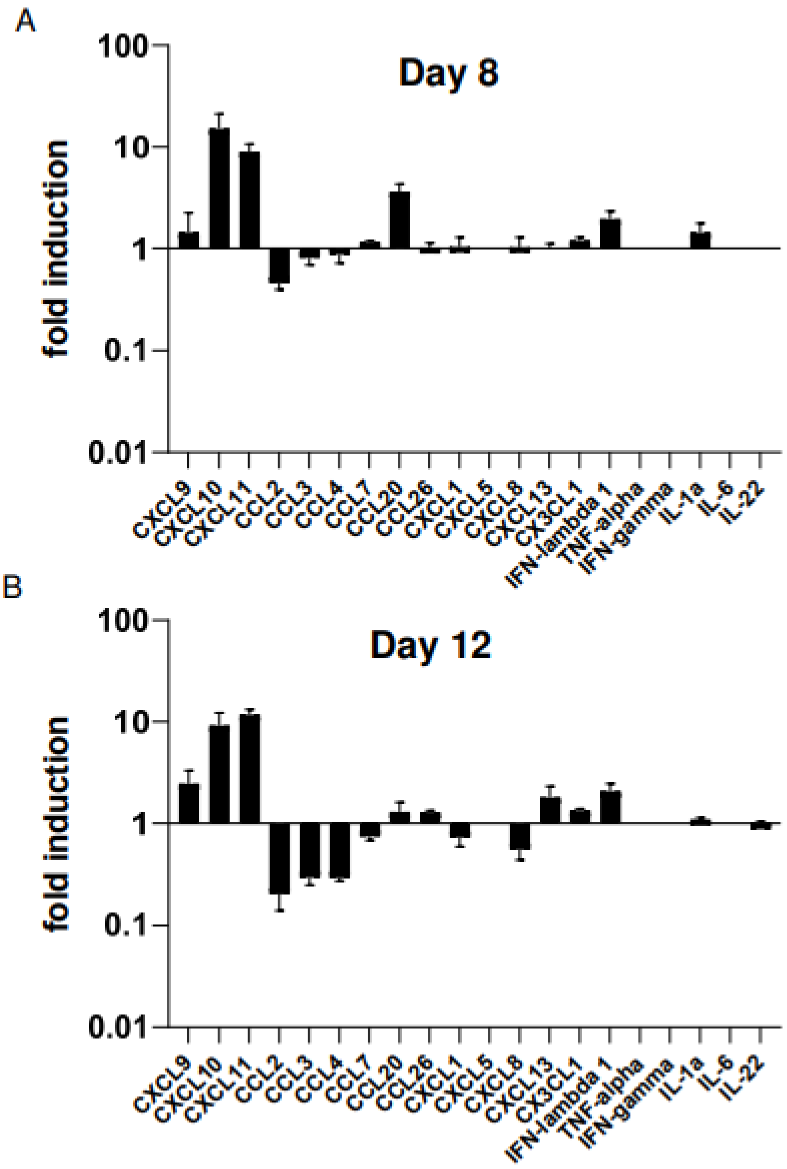

Fig. S3: Relative induction of secreted proteins in the supernatants of HDV mono-infected vs uninfected PHHs measured on the Luminex platform (**A)** 8 days and **(B**) 12 days after HDV infection. Boxes represent mean, vertical lines represent standard deviation.

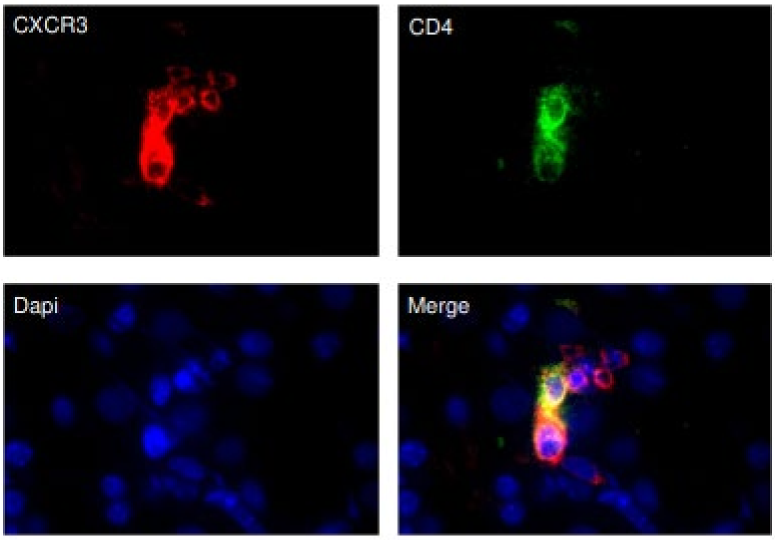

Fig. S4: CXCR3 (red)- and CD4 (green)- positive cells in frozen liver sections of HBV/HDV-infected chimeric mouse livers receiving HBsAg-specific T cells were analyzed by IF.
